# Lower Energy-Dense Ready Meal Consumption Affects Self-Reported Appetite Ratings with No Effect on Subsequent Food Intake in Women

**DOI:** 10.3390/nu13124505

**Published:** 2021-12-16

**Authors:** Sophie C. Hannon, Sarah E. Hillier, Pariyarath S. Thondre, Miriam E. Clegg

**Affiliations:** 1 Oxford Brookes Centre for Nutrition and Health, Faculty of Health and Life Sciences, Oxford Brookes University, Oxford OX3 0BP, UK; sophie.hannon65@hotmail.com (S.C.H.); sarah.hillier@solent.ac.uk (S.E.H.); pthondre@brookes.ac.uk (P.S.T.); 2 Faculty of Sport, Health and Social Sciences, Solent University, Southampton SO14 0YN, UK; 3 Institute for Food, Nutrition and Health, Department of Food and Nutritional Sciences, University of Reading, Reading RG6 6DZ, UK

**Keywords:** appetite, satiety, ready meals, energy density, food intake

## Abstract

Slimming World (SW), a commercial weight management organisation, has designed a range of low energy-dense ready meals (LEDRMs) in line with their programme. This randomised crossover study compared commercially available equicaloric ready meals differing in energy density on satiety and food intake. It was hypothesised that the LEDRM would reduce energy intake (EI) whilst increasing fullness and reducing hunger compared to higher energy-dense ready meal (HEDRM, control). A total of 26 female participants (aged 18–65 years; body mass index of 28.8 ± 3.0 kg·m^−2^) attended two test days. The participants ate a standard breakfast, and four hours later, ate either a LEDRM or HEDRM at lunch. EI was measured four hours later at an *ad libitum* tea. Satiety measurements were recorded throughout the day using visual analogue scales and a weighed food diary was completed for the remainder of the day. The results revealed that the LEDRM reduced hunger and increased fullness (both *p* < 0.001). There was no difference in EI at the evening meal between the ready meals (*p* > 0.05), however, during the whole LEDRM testing day, the participants consumed significantly less fat (7.1%) and saturated fat (3.6%) (both *p* < 0.01), but significantly more carbohydrates, sugars, fibre, protein, and salt (all *p* < 0.01). The results indicate that the participants felt more satiated after consuming ready meals of the same energy content but larger portion size. Despite no significant difference in short-term EI between the ready meals, the results indicated that the LEDRM produced beneficial subjective satiety responses and, therefore, can help to improve the nutritional content of meals i.e., reduce saturated fat consumption.

## 1. Introduction

Overweight (body mass index [BMI] ≥ 25 kg·m^−2^) and obesity (BMI ≥ 30 kg·m^−2^) have become global concerns to public health [1]. In England, statistics indicated that 64% of adults were overweight or obese in 2019, with men being more likely to be overweight than women (41% vs. 31%, respectively), but women more likely to be obese compared to men (29% vs. 27%, respectively) [2]. Overweight and obesity can increase an individual’s risk of developing diseases, such as cardiovascular diseases, type two diabetes, osteoarthritis, and many forms of cancers [1]. One of the major causes of Overweight and obesity is an excessive consumption of energy, beyond what is utilised by the body [3,4]. The current guidelines to reduce obesity suggest diet and lifestyle alterations [5]. Commercial weight management organisations have been able to help the public manage their diet and make lifestyle changes to reduce calorie intake, helping with weight management [6]. Furthermore, in a study by Crane et al. [7], 31.2% more females reported having utilised an organised weight loss program during a period of weight loss compared to males.

In the UK, Slimming World (SW) is classified as a tier 2 weight management programme that uses evidenced-based behaviour change techniques to encourage members to adopt healthier lifestyles, by supporting changes in eating and physical activity behaviours. The dietary component of the programme, referred to as ‘Food Optimising’ (for full SW programme information see Slimming World [8]), places an emphasis on the *ad libitum* consumption of low-energy-dense (LED) foods, e.g., lean meats, eggs, fish, pasta, fruits, and vegetables, referred to as ‘Free Food’. Energy density refers to the number of calories per gram of a food (kcal·g^−1^) [9]. SW also advocates for controlled amounts of foods with high energy density to support a decrease in energy intake, whilst aiming to strengthen sensations of satiation and satiety [10]. Previous research suggests that consuming LED foods can reduce hunger sensations and subsequent meal energy intake compared with higher ED foods in healthy weight, overweight and dieting individuals [11,12,13].

In 2015, SW released a range of frozen ready meals with Iceland Food Ltd., to be a convenient option for its members when cooking from scratch was not feasible. These ready meals were designed to be in line with the dietary principles of the programme, focusing on reducing EI, both within and between meals, in order to diminish the effect of hunger [8]. Ready meals, defined as ‘main courses that can be reheated in their container, needing only minimal preparation before consumption’, are a vastly profitable sector of the food industry [14]. The current statistics report that 93% of UK adults consume ready meals, with families being regular users [15]. In 2018, the UK ready meal market entered its fourth year of growth, with the estimated forecast growth expected to continue over the next five years, with time-scarcity deemed a major motivation for their use [15,16]. Alkerwi et al. (2015) found that daily consumption of ready meals was associated with a higher energy intake (EI) and poor compliance to national nutritional guidelines; specifically, high fat intakes and low dietary fibre intakes [17]. Additionally, there was a positive association between ready meal consumption and central obesity [17].

Considering the projected forecast increase in ready meal consumption, it is important to understand how ready meal consumption might influence satiety [15]. It is valuable to understand the satiating properties of ready meals, specifically in the context of weight management meals, in order to establish if they have an impact on EI. With women being more likely to take part in organised weight loss programmes [7], the authors deemed it to be of greater relevance to conduct this investigation on women only. To the knowledge of the authors, no studies have investigated the impact these products have on satiety amongst overweight women.

This research aimed to explore the effects of two ready meals available for sale on the UK market on subjective short-term satiety ratings and subsequent energy and macronutrient intake among women with a BMI ≥ 25 kg·m^−2^. Using a pragmatic approach, two ready meals were served in their respective one-person serving sizes. The meals differed in energy density (ED) (low-energy-dense ready meal; LEDRM and a high-energy-dense ready meal; HEDRM), and consequently differed in their macronutrient and fibre composition, but had a similar total energy composition. It was hypothesised that the LEDRM would reduce EI whilst increasing fullness and reducing hunger, desire to eat (DTE), and prospective consumption ratings compared to the HEDRM.

## 2. Materials and Methods

A within-subjects, single-blind, randomised crossover design was used to examine the effects of consuming equicaloric ready meals differing in ED at lunch. The trial methodology was retrospectively registered at ClinicalTrials.gov (NCT04994925) and conducted between March and October 2019. Self-reported subjective appetite ratings were measured and subsequent food intake during an *ad libitum* buffet meal and food intake for the remainder of the day were recorded using a weighed food diary. The participants were required to visit the Oxford Brookes Centre for Nutrition and Health (OxBCNH) for one screening session and two test sessions. There was a minimum 24-h washout period between test sessions to reduce the likelihood of any carry-over effect [18].

The ready meals investigated were a LEDRM lasagne (Slimming World Free Food Beef Lasagne 550 g, Deeside, UK) (the test meal) and a HEDRM lasagne (Supermarket brand Beef Lasagne 440 g, London, UK) (the control meal) (Table S1). The ready meals differed in weight (Table 1; Control = 380 g & Test = 538 g). Lasagne was the chosen ready meal because it was possible to find a comparative higher energy dense lasagne ready meal available on the market. Evaluation of liking was conducted on the ready meals in a separate sample of participants.

Based on preliminary data [19] with an alpha level of 0.05 and statistical power of 0.8, it was estimated that a sample size of 25 female participants would be required to detect a 331 kcal difference in total daily energy intake (TDEI).

**Figure 1:**
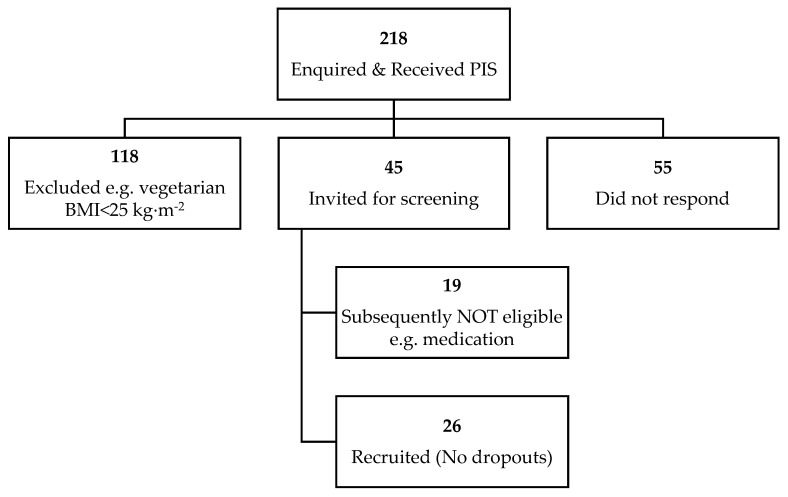
Flow diagram illustrating participant recruitment.

All individuals who enquired (*n* = 218, Figure 1) received a participant information sheet (PIS), which informed them of the research details. Participants were healthy, non-smoking women (aged 18–65 years with a BMI ≥ 25 kg/m^2^), who met the following inclusion criteria: no known food allergies to the study foods, no eating disorders, not following a special diet (e.g., vegetarian, halal, actively dieting or on a weight loss diet), not taking any medication or supplements known to affect appetite or weight within the month prior to and/or during the study, not pregnant, planning to become pregnant or breastfeeding, having no significant change in their physical activity in the 2–4 weeks prior to the study, not receiving systemic or local treatment likely to interfere with the evaluation of the study parameters, not had a gastric band/had undergone gastric bypass treatment or worked in appetite or feeding related areas.

Twenty-six female volunteers were recruited via posters placed around Oxford Brookes University campuses, social media platforms (Twitter, Instagram, LinkedIn), local newspaper advertisements, local community council notice boards, attending local SW group meetings, and word of mouth between April 2019 and October 2019.

All eligible participants visited the OxBCNH for a screening visit, in which written informed consent was obtained first. During this screening visit, participants were also asked if there were any foods they would or could not eat. Subsequently, each participant’s height was measured using a fixed stadiometer (SECA 264, Hamburg, Germany) to the nearest 0.1 cm according to The International Society for the Advancement of Kinanthropometry standards [20]. Next, body composition was measured using bioelectrical impedance (Tanita, BC-418MA, Amsterdam, The Netherlands), providing information on weight (kg), BMI (kg/m^2^) and body fat percentage (%). Ethical approval for the study was obtained from the Faculty of Health and Life Sciences at Oxford Brookes University (DREC reference 0119_44) in accordance with the guidelines laid down in the Declaration of Helsinki. All participants had the opportunity to discuss the PIS and ask any questions regarding the study protocol/procedure prior to providing written informed consent. Upon completion of both test days and returning the food diaries, participants received a £15 Amazon voucher for compensation of their time.

Although participants were aware the study was investigating satiety, the order of each ready meal for each participant was randomised using an online uniform distribution randomiser (www.randomizer.org; accessed on 26 February 2019). The participants were also blinded to which ready meal was being provided.

### 2.1. Overview of Study Protocol

**Figure 2:**
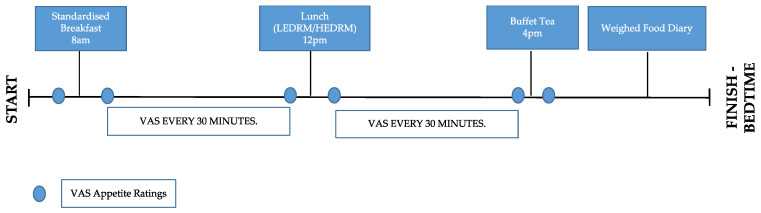
Timeline of study protocol for both test days.

The protocol was repeated twice; once with the test meal (LEDRM) and once with the control meal (HEDRM). There were four hours between each meal and the mealtimes (Figure 2) were determined by the time participants began breakfast. Participants were instructed to remain in the building and refrain from eating between mealtimes.

#### 2.1.1. Pre-Trial Standardisation

Participants received a reminder email 24 h before commencing the study, instructing them to fast for 12 h prior to their trial start time, to limit caffeine intake (maximum two/three cups of tea, coffee and/or caffeinated soft drinks) and avoid alcohol and strenuous exercise [21]. Compliance was confirmed by the researcher. Participants were not required to standardise their diet the day before the study due to the period of fasting and the standard breakfast being received by each participant before the ready meals were consumed.

#### 2.1.2. Breakfast

Breakfast was standardised for each participant for both test sessions. The energy content of the breakfast (400 kcal) was based on 20% of the standard UK female total daily energy requirements as recommended by Public Health England [19]. Participants were required to consume the entire contents of the breakfast, which included toast, jam, margarine, and a choice of cereal (Alpen No Added Sugar Swiss Style Muesli, Nestle Cheerios or Special K Original, UK). Black tea (250 mL) or coffee (250 mL) were provided if desired and any milk added to the beverages came from the 160 mL provided. This was replicated on both test days.

Water consumption was provided *ad libitum* during the first test day and was replicated for mealtimes during the second test day. The volume of water consumed between mealtimes on each test day was not standardised due to the free-living conditions, and it being deemed unethical by the research team to not allow the participants to consume water when thirsty. Furthermore, there is research indicating that water served alongside food does not affect subsequent energy intake [22]. Participants were instructed not to consume a large quantity of water prior to mealtimes to try and negate any potential differences in gastric distension and subsequent appetite responses between the test days.

#### 2.1.3. Lunch

The ready meals were given to the participants in the portion size in which they were bought from the manufacturer. They were served in their original tray packaging on a standardised white dinner plate. A green salad was provided (24 g = average serving) alongside the lasagne, as SW advocate consuming a vegetable or salad with their ready meals [23]. The participants’ meals were weighed on scales (Mettler PC 2000, Greifensee, Zurich, Switzerland) to the nearest 0.1 g and recorded by the researcher before being served and participants were required to eat each meal in its entirety.

Participants were able to take as long as was required to finish each meal, but they were not allowed to undertake any activity that would distract their focus from the task of eating (e.g., using electronic devices, reading). All of the meals were prepared in the OxBCNH kitchen and dispensed to the participants using safe food hygiene measures. All eating utensils provided to each participant were standardised throughout.

#### 2.1.4. Buffet Tea

A buffet tea was chosen to measure food intake in order to provide the participants with a choice of foods, purposefully in abundance, and in an environment where the quantity of foods consumed could be measured. The *ad libitum* buffet tea included a selection of yogurts, cereal snack bars, fruit, vegetable sticks, and sandwiches with participants having chosen three from the six sandwich options (including chicken, tuna, ham, beef, egg, and houmous, with two portions of each option, equaling six in total). Participants were made aware that they were able to request more of anything should they have wanted. Participants were instructed to eat until they felt comfortably full, at which point the buffet tea was terminated. Once the participant had finished eating, the researcher re-weighed and recorded any food that had been left by the participant.

#### 2.1.5. Remainder of Test Day

Participants were required to complete a weighed food diary for the remainder of the test day for both sessions. The researcher provided explicit information on how to complete the food diary and written instructions were provided for reference. Participants were provided with digital scales (Argos Home Digital Kitchen Scale, UK) and a food diary to complete until they went to sleep at the end of the test days.

### 2.2. Outcome Measures

#### 2.2.1. Visual Analogue Scale (VAS) Measurements

Subjective appetite ratings for hunger, fullness, desire to eat (DTE), prospective food intake, thirst, and nausea were measured by drawing a vertical line through six separate 100 mm (mm) VAS measurements, which have been validated and are considered a reliable measurement for subjective appetite sensations [24]. Ratings were made prior to the breakfast (fasted), then every 30 min from commencing breakfast (8 a.m.) until lunch (12 p.m.). VAS ratings continued every 15 min from commencing lunch until the buffet tea (4 p.m.), with the last rating being made after finishing the buffet tea. Participants were required to complete a sensory analysis (attractiveness, smell, texture, aftertaste, tastiness, eat again, and pleasantness) of the lunch meals immediately after consuming them as it is known that palatability can influence subjective appetite sensations [25,26]. VAS ratings were quantified by measuring the distance from the anchor on the left side of the line to the vertical line the participants had marked, using a ruler, to the nearest mm [9].

#### 2.2.2. Food Intake

The energy, fat, saturates, carbohydrates, sugars, fiber, protein, and salt content of the food consumed during the buffet tea and weighed food diaries was calculated using the nutritional composition from the label of each ingredient by inputting into Nutritics software (Nutritics Ltd., Dublin, Ireland).

### 2.3. Statistical Analysis

All statistical analyses were performed on Statistical Package for the Social Sciences (SPSS, version 24.0; IBM Corp, Armonk, NY, USA). Data were tested for normality using the Shapiro–Wilk test of normality. Differences in food intake between the ready meals (during the buffet tea, food diary and combined) and differences between subjective sensory evaluation ratings between ready meals were analysed using a paired samples t-test for normally distributed data (i.e., parametric data) and the Wilcoxon matched pairs signed-rank test for not normally distributed data (i.e., non-parametric data). The areas under the curve (AUC) from self-reported appetite ratings from VAS (hunger, fullness, DTE, prospective consumption, thirst, and nausea) were calculated using the trapezoid rule. If data were non-parametric, they were log transformed and then a univariate ANOVA using the baseline VAS as a covariate in the analysis was completed to assess differences between the meals. If data were parametric, they were not log transformed. Statistical significance was accepted at *p* < 0.05. Data are presented as means ± standard deviation.

## 3. Results

All of the recruited participants (*n* = 26, aged 40 ± 15 y, height 1.63 ± 0.07 m, weight 76.7 ± 10.3 kg, BMI 28.8 ± 3.0 kg/m^2^) completed both test days in a within-subject crossover design.

### 3.1. Self-Reported Appetite Ratings

There were no significant differences in the appetite ratings after consuming the standardised breakfast between the test days (*p* > 0.05). Thus, suggesting that the standardised breakfast was effective in standardising the appetite ratings between the testing days.

**Figure 3:**
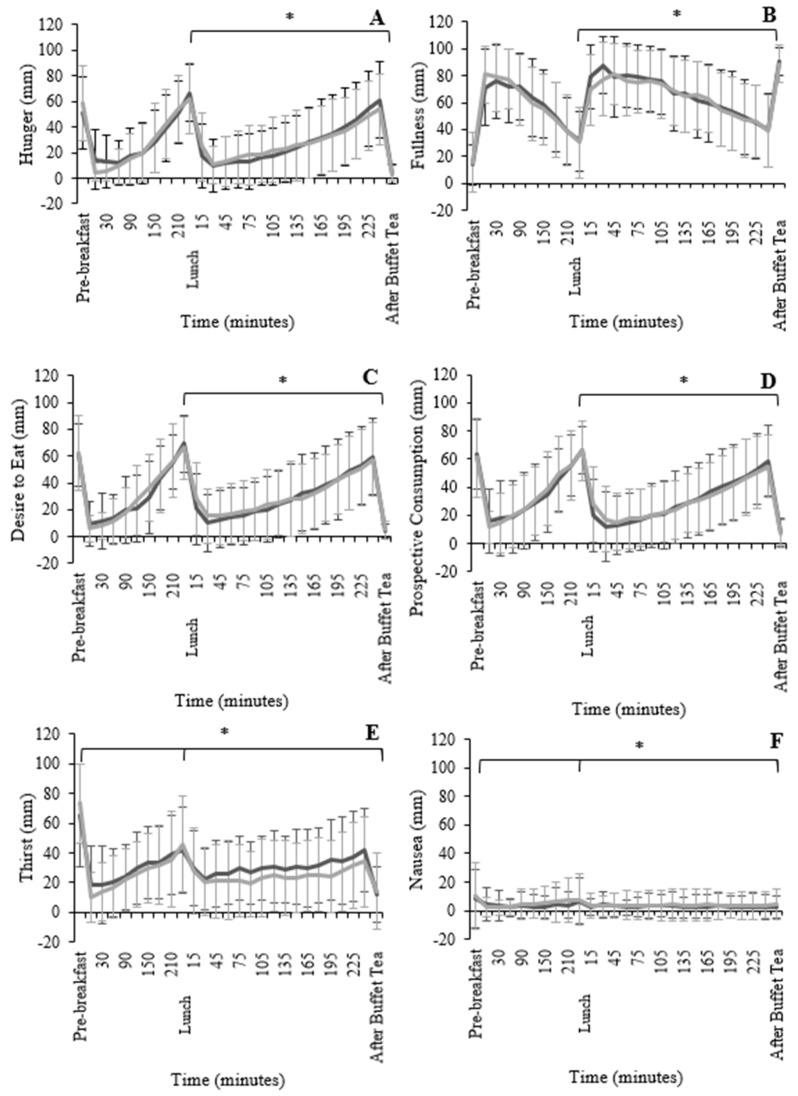
Mean (±standard deviation) for hunger (**A**), fullness (**B**), desire to eat (**C**), prospective consumption (**D**), thirst (**E**), and nausea (**F**) from VAS taken throughout the HEDRM test day (black) and the LEDRM test day (grey). Significance tested by analysis of variance (ANOVA) using baseline values as covariate. * Indicates significant difference between test meals.

The total AUC for the whole test day (including breakfast, lunch, and tea) indicated that there was no significant difference in hunger (F_1,49_ = 0.59, *p* = 0.56; Figure 3A), fullness (F_1,49_ = 2.00, *p* = 0.15; Figure 3B), DTE (F_1,49_ = 0.49, *p* = 0.62; Figure 3C), and prospective consumption (F_1,49_ = 0.01, *p* = 0.99; Figure 3D) between the two ready meals. However, data analysed from the time the ready meals were consumed (lunch) until the buffet tea, indicated that the participants felt significantly more hunger (F_1,49_ = 6.24, *p* < 0.001), had a greater DTE (F_1,49_ = 8.47, *p* = 0.004), and greater prospective consumption ratings (F_1,49_ = 7.09, *p* = 0.001) after consuming the HEDRM when compared to LEDRM. Conversely, fullness (F_1,49_ = 2.55, *p* < 0.001) ratings from lunch until the buffet tea were significantly greater for the LEDRM compared to the HEDRM.

Thirst was significantly greater (*p* < 0.01) for the whole HEDRM day compared with the LEDRM day. Thirst between the lunch and the buffet tea was significantly greater (F_1,49_ = 41.8, *p* < 0.001; r^2^ = 0.615) after the LEDRM compared to after the HEDRM (Figure 3E). The nausea ratings for the whole day were significant (F_1,49_ = 20.6, *p* < 0.01; r^2^ = 0.434), with participants’ measurements indicating greater nausea after the HEDRM compared to the LEDRM. The nausea ratings between the lunch and the buffet tea were significant (F_1,49_ = 22.7, *p* < 0.001; r^2^ = 0.459), indicating that nausea was greater after the HEDRM compared with the LEDRM (Figure 3F).

### 3.2. Sensory Analysis

**Figure 4:**
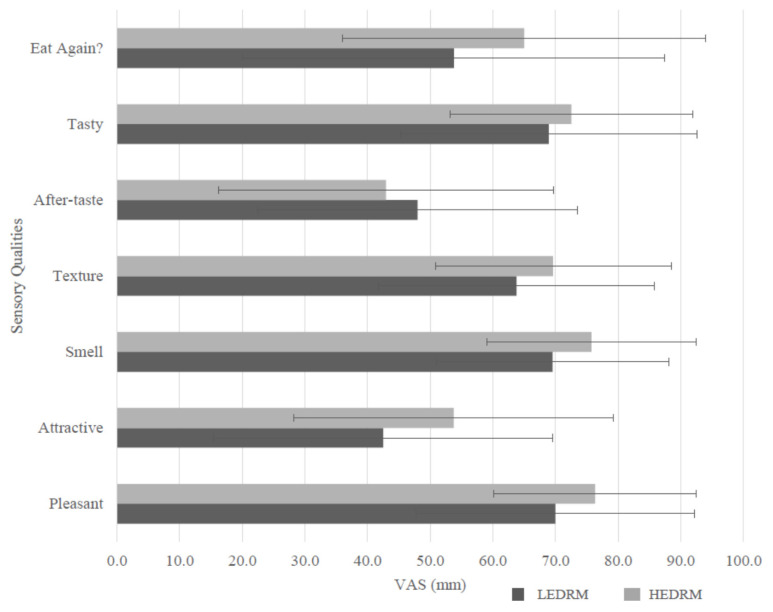
Participant evaluation of the low-energy-dense ready meal (LEDRM) and high-energy-dense ready meal (HEDRM) for the following attributes: attractiveness, smell, texture, after taste, tastiness, eat again and pleasantness. Significance tested using paired samples *t*-test or Wilcoxon signed-rank test.

The attractiveness was significantly higher in the HEDRM compared with the LEDRM, (d = 0.585; *p* = 0.01), with no significant difference (*p* > 0.05) between the other six sensory characteristics (smell, texture, after taste, tasty, eat again, pleasant; Figure 4).

### 3.3. Food Intake

**Figure 5:**
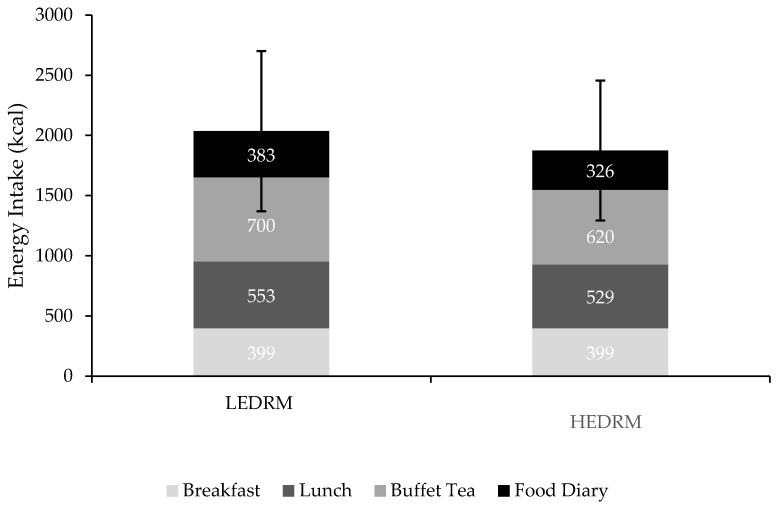
Mean energy intake (kcal) from breakfast, test lunch, buffet meal, and food diary during the low-energy-dense ready meal (LEDRM) test day and the high-energy-dense ready meal (HEDRM) test day.

There was no significant difference in EI during the buffet tea after the LEDRM or HEDRM (*p* > 0.05; Figure 5; Table 2). The participants consumed significantly more saturated fat (r = 0.319; *p* = 0.02) and carbohydrates, of which sugars (r = 0.298; *p* = 0.03), at the buffet tea during the LEDRM day compared to the HEDRM day. There was no significant difference in total fat, carbohydrates, fibre, protein, and salt intake between the two buffet teas (*p* > 0.05). There was no order effect during the buffet tea between either of the testing days (*p* > 0.05).

TDEI for the whole testing days (breakfast, lunch, buffet tea, and weighed food diary) was tending towards significance, albeit non-significant, for the LEDRM day (~2035 kcal) compared to the HEDRM (~1874 kcal) (*p* = 0.06; total difference = 161 kcal). The participants consumed significantly less fat (r = −0.403; *p* = 0.004) and saturated fat (r = −0.537; *p* < 0.001) during the whole LEDRM day compared to the whole HEDRM day. The total carbohydrate (d = 0.810; *p* < 0.001), sugar (r = 0.456; *p* = 0.001), fibre (r = 0.572; *p* < 0.001), protein (r = 0.474; *p* = 0.001), and salt (r = 0.305; *p* = 0.028) intake between the two test days was significantly greater during the LEDRM day compared to the HEDRM day (*p* < 0.01).

## 4. Discussion

The purpose of the present study was to investigate the effects of two ready meals currently available on the UK market that differed in ED on self-reported short-term satiety ratings and subsequent dietary intake among women with a BMI ≥ 25 kg/m^2^. It was hypothesised that after consuming the LEDRM, subjective ratings for hunger, DTE and prospective consumption would decrease, whilst ratings of fullness would increase compared to after the HEDRM. Additionally, it was hypothesised that EI would be reduced during the LEDRM testing day, based on previous evidence by Rolls et al. [11] that suggests consuming LED foods can reduce hunger sensations and subsequent meal energy intake compared with higher ED foods in overweight individuals. The main findings supported the proposed hypothesis with regards to the subjective appetite sensations; fullness increased whilst hunger, DTE and prospective consumption decreased after the LEDRM. However, there was no significant difference in EI following the two ready meals for both the buffet tea and the whole day intake. These results indicate that the LEDRM may aid in subjective appetite regulation but that this might not translate, at least in the short-term, to a reduction in total EI. Although the whole day intake showed no significant difference between the two test days, it should be noted that the use of food diaries may cause participants to alter food intake with evidence to suggest potential underestimation on EI [27].

The decrease in the subjective appetite ratings reported in the current study is supported by the findings from Buckland et al. [13], who found that women with overweightness or obesity reduced hunger, DTE, and prospective consumption, whilst increasing fullness when they consumed low-energy-dense (LED) meals compared to high-energy-dense (HED) meals. These subjective appetite ratings were established throughout the whole day of testing, unlike in the current study, with differences in appetite ratings only being found between the lunch and the buffet tea. It could be suggested that this occurred due to the breakfast in the current study being identical (unlike LED and HED breakfasts provided in the Buckland study) and so no differences in appetite ratings were expected during the breakfast and lunch time points in the current study. It must be acknowledged that the current study did not restrict the recruitment of participants to habitual breakfast eaters only and provided standardised breakfast portions only as this may have reduced the final number of participants recruited. Recruiting a mixture of habitual and non-habitual breakfast eaters could have led to enhanced feelings of fullness in some participants, as the energy provided at breakfast was not tailored to individual’s daily energy need.

Despite the similar appetite results from this study and Buckland et al. [13] there was no similarity in EI between the two studies. The *ad libitum* evening EI and TDEI was significantly reduced on the LED day versus the HED day in the Buckland et al. [13] study, whereas there was no difference in buffet tea EI and TDEI between the LEDRM and HEDRM in the current study. Considering the current study was conducted on ready meals only, and although the difference in ED was minor (0.32 kcal/g), it could be suggested that in order to achieve any impact on TDEI, a greater disparity is required in the difference between the low and high ED meals consumed (1.7 kcal/g difference in Buckland et al. [13]). Furthermore, it may be necessary for all meals to be LED throughout the day—not simply just one meal—for there to be an effect on EI, which could subsequently impact on weight loss. It should also be noted that the women in the Buckland et al. [13] research were actively engaged in weight loss, which was not the case for all participants in the current study [13]. Thus, it appears possible that active weight loss is not necessarily required for the impact of LED foods on acute appetite ratings to be realised, however, it may be a factor in establishing a difference in calorie intake and potentially weight loss over time. Long-term reduction in ED without reducing the weight of the food consumed can beneficially impact the BMI of individuals aiming to lose weight [28]. Further research warrants investigation into the implication of long-term ready meal consumption and the impact this may have on energy balance and weight.

Results from a recent systematic review and meta-analysis investigating the effect of energy density on appetite found fullness to be increased after consuming high-energy-dense diets compared to low-energy-dense diets [29]. This differs from the current study in which fullness increased after the LEDRM. However, it is important to note that the intervention periods of the studies included in the review varied from one hour to several days and therefore it is difficult to extrapolate these results and compare them to the current study, which was short-term [29]. In the current study, hunger decreased after the LEDRM, whereas there was no reported difference between the HED, and LED diets included in the systematic review [29]. However, it is important to note that the HED diet was ~1.65 kcal/g in the systematic review [13] whereas the current study, the HEDRM was 1.38 kcal/g (0.27 kcal/g difference). Furthermore, a study by Pritchard et al. [30] found no difference in hunger or fullness between HED and LED cottage pie, whereas the current study and Buckland et al. [13] did report differences between the energy-dense meals. A possible explanation for the differences in study outcomes could be due to the differing effects each macronutrient within each meal has on satiety and appetite.

The participants in this study were provided with standard ‘shop-bought’ portion sizes, which differed in weight (difference of 150 g). It is credible that the packaging of the two ready meals might have influenced satiety responses, as the LEDRM was provided in a foil container whereas the HEDRM was in a plastic container and was visibly smaller in size. This may have caused the individuals to modify subjective perceptions of satiety signals when consuming each ready meal [31]. A study conducted by Rolls et al. [32] served participants varying portion sizes of macaroni cheese, finding participants consumed 30% more energy when offered the largest portion than when given the smaller portion. Despite these differences in visible portion size, subsequent subjective ratings of hunger and fullness did not differ. Conversely, in Rolls et al. (2004) where portion size of a snack of crisps was increased, fullness ratings increased with the increased crisp portion size [11]. It is possible that this was affected by a visual indication but also highlights the issues with comparing varying study designs within appetite research. In the current study, it is important to note that the two ready meals were provided on separate days. The LEDRM was visibly larger, however, the participants may have forgotten this as they did not directly compare meals on the same testing day.

It should also be noted that attractiveness from the sensory evaluation of the ready meals was significantly greater for the HEDRM compared to the LEDRM. Considering sight is encompassed within the sensory aspect of the satiety cascade, it could be suggested that this would contribute to satiety [33]. However, in the current study this did not appear to influence the appetite ratings or food intake, as the LEDRM had reduced hunger and increased fullness compared to the HEDRM. As mentioned, it is important to note that these meals were evaluated on different days and as such, attention should be given when interpreting these results as this may have influenced the sensory evaluation [34].

As the weight of the LEDRM was greater than the HEDRM (+150 g), gastric distension may have affected vagal afferents [35] i.e., the signaling along the vagus nerve of the gut-brain axis to liaise to the brain that the individual is full, to a greater extent. Results from Wang et al. [35] suggest that gastric distension is more important in ratings of fullness compared to hunger. The current study supports this research regarding subjective fullness feelings being impacted by greater distension. This may have been because the LEDRM was greater in weight and volume, perhaps causing greater gastric distension compared to the HEDRM, consequently increasing feelings of fullness. However, EI at the buffet tea was not significantly less for the LEDRM day despite the increase in the feelings of fullness. It is hypothesised that the increase in mass of the LEDRM caused changes in subjective feelings of fullness but not in the feedback loop to subsequently impact EI in the four-hour testing period. However, gastric distension was not measured and so this is difficult to clarify.

There was more protein and fibre (44% and 69%, respectively) in the LEDRM compared to the HEDRM. Protein and fibre have satiation enhancing effects [36], therefore, they may have contributed to increased satiety following the LEDRM. The differences in satiety responses did not impact subsequent EI, which suggests that people may override signals when provided with a large proportion of food i.e., during the buffet tea. Data from the American Institute of Cancer Research (2004) found that 69% of adults (*n* = 1000) would finish their meals when dining out, of which 30% stated that they would have been satisfied with a smaller portion [37]. Thus, in the current study, the buffet tea food intake may have been affected by the appeal of free food, including potential novel products not usually consumed [20]. A buffet is one the most effective ways of evaluating food intake objectively under controlled settings but may not necessarily represent what individuals would choose for themselves outside of the study environment and may not be representative of a typical evening meal. Participants may have consumed food in the absence of hunger or ignored satiety signals, which could have impacted the relationship between appetite ratings and EI between the testing days, ultimately limiting external validity [33]. Further research could utilise a methodology in which participants could choose when they begin their buffet tea meal. This would mimic more ‘real-life’ setting and help to determine the influence of set mealtimes on satiety.

While the researchers aimed to maintain similar energy in the ready meals used, this was difficult to achieve because the study was investigating ready meals that were commercially available on supermarket shelves and, as such, were the best calorie-matched ready meals at the time of research (difference of 23 kcal/portion). As such, this study was a pragmatic approach of assessing differing ED with ready meals in the UK supermarkets at the time of research. Although there appeared to be no significant difference in calorie intake between the days and thus there is no indication of altering energy balance, the participants’ macronutrient intake (specifically fat) was reduced on the LEDRM day compared to the HEDRM day [38,39]. This is due to the LEDRM day being lower in total fat and saturated fat, and higher in fibre and protein. However, it is important to note any conclusions drawn from this study are only relevant within the context of consuming lasagne as no other ready meals were used. It should also be noted that lasagne may not be habitually consumed during a lunchtime meal. As such, further research warrants exploration into the potential effects of differing ready meals. It may also be beneficial to continue this study on individuals who rely heavily on ready meals habitually and determine how outcomes may impact body weight over a longer time frame.

In the current study, the effect of ready meal consumption on appetite and satiety was only explored within the context of women, due to women partaking in weight loss diets to a greater extent than men [7]. Thus, the results intrinsically have an all-female bias. Considering only women were included in the current study, it would be beneficial in the future to consider how sex hormones may potentially impact satiety ratings and EI during different phases of the cycle [40,41]. A study by Brennan et al. [42] found stunted gastric emptying rates reduced subjective hunger ratings, which may have influenced the reduced EI, depress plasma insulin, glucose, and GLP-1 concentrations during the follicular phase of the cycle relative to the luteal phase. Thus, indicating women should be tested during the same phase of their menstrual cycle to minimise any hormonal impact on satiety ratings or EI.

## 5. Conclusions

The current research suggests that LED, calorie-matched ready meals are favourable for subjective appetite ratings, aiding appetite control, but that there is no impact on TEI.

Although the LEDRM buffet tea intake contained 0.8 g more saturated fat compared to the HEDRM buffet tea intake, analysis of the whole test day showed that total fat and saturated fat content were significantly reduced on the LEDRM day compared to the HEDRM day. As such, it may be beneficial for food manufacturers to continue to provide LEDRM to increase fullness and lower consumer saturated fat intake, potentially impacting health, especially for those who may habitually consume ready meals. With the continued growing ready meal market, it is important for further research using longitudinal studies to investigate if the enhanced satiety benefits of the LEDRM translate into changes in EI and subsequently body weight.

## Figures and Tables

**Table 1:** Nutritional values for test low-energy-dense ready meal (LEDRM) and control high-energy-dense ready meal (HEDRM) per 100 g and per portion (550 g for LEDRM and 400 g for HEDRM) as well as the salad leaves that accompanied the meals and the total for each test meal.

Nutrients	Test LEDRM		Control HEDRM		Salad Leaves	Total Meal	
	Per 100 g	Per 550 g	Per 100 g	Per 400 g	24 g	LED + Salad	HED + Salad
Energy (kJ)	430	2313	575	2199	17	2330	2216
Energy (kcal)	102	549	137	525	4.0	553	529
Fat (g)	2.1	11.3	5.7	21.8	0.5	11.8	22.3
Saturates (g)	0.8	4.3	2.5	9.7	0.1	4.4	9.8
Carbohydrates (g)	10.7	57.6	11.6	44.5	0.5	58.1	45.0
Sugars (g)	1.6	8.6	2.6	10.1	0.5	9.1	10.6
Fibre (g)	1.7	9.1	1.4	5.4	0.5	9.6	5.9
Protein (g)	9.3	50.0	9.1	34.8	0.5	50.5	35.3
Salt (g)	0.4	2.2	0.4	1.6	0.0	2.2	1.6

**Table 2:** Nutritional composition of food intake during the buffet meal and during the whole test days (breakfast, lunch, buffet tea and weight food diary combined) during the low-energy-dense ready meal (LEDRM) test day and the high-energy-dense ready meal (HEDRM) test day.

	LEDRM Buffet Tea	HEDRM Buffet Tea	LEDRM Whole Day	HEDRM Whole Day
Energy (kJ)	2931 ± 2302	2605 ± 2302	8359 ± 2759	7859 ± 2452
Energy (kcal)	700 ± 570	620 ± 544	2035 ± 667	1873 ± 582
Fat (g)	11.1 ± 10.7	9.7 ± 10.5	45.8 ± 23.3	57.0 ± 18.5 †
Saturates (g)	3.5 ± 3.4 †	2.7 ± 3.1	14.9 ± 5.1	21.2 ± 7.6 †
CHO (g)	100.2 ± 82. 0	88.4 ± 76.8	260.0 ± 81.5 †	220.3 ± 76.4
Sugars (g)	38.6 ± 29.1 †	31.4 ± 26.4	91.0 ± 35.0 †	76.2 ± 28.7
Fibre (g)	15.6 ± 11.7	14.3 ± 11.6	35.1 ± 13.1 †	28.0 ± 11.5
Protein (g)	39.6 ± 29.7	34.9 ± 30.7	119.2 ± 28.3 †	101.0 ± 32.4
Salt (g)	2.4 ± 2.0	2.1 ± 1.8	6.5 ± 2.2 †	5.6 ± 1.9

CHO = carbohydrate † indicates significance (*p* < 0.05). † significance tested using Wilcoxon signed-rank test.

## Data Availability

Data can be made available upon request to the authors.

## Supplementary Materials

The following are available online at https://www.mdpi.com/article/10.3390/nu13124505/s1, Table S1: Test meal lunch list of ingredients.
