# Supplementary material for: Lower Energy-Dense Ready Meal Consumption Affects Self-Reported Appetite Ratings with No Effect on Subsequent Food Intake in Women
Source: Nutrients. 2021 Dec 16;13(12):4505. doi: 10.3390/nu13124505 (PMC8708992; doi:10.3390/nu13124505)
Supplement: Supplementary file 1 [file nutrients-13-04505-s001.zip › nutrients-1463720-supplementary.pdf]

Supplementary material Table S1: Test meal lunch list of ingredients

	Ingredients
Slimming World Lasagne	Cooked minced beef (25%), water, pasta sheets (11%) (durum wheat semolina, water, wheat flour (wheat flour, calcium carbonate, iron, niacin, thiamin), dried whole egg, dried egg white), chopped tomatoes (9%) (tomatoes, tomato juice), tomato paste, sliced mushrooms, quark (skimmed milk soft cheese), diced onion, Worcester sauce (water, white vinegar, sugar, salt, tamarind extract, onion powder, barley malt extract, garlic powder, ground ginger, concentrated lemon juice, clove powder, chilli powder), butternut squash, potato, diced carrot, diced celery, beef bouillon (yeast extract, potato starch, salt, flavouring, chicory extract, beef stock (beef extract, onion powder, carrot extract, tomato powder, dried lovage), garlic purée, herbs, maize starch, vegetable bouillon (yeast extract, salt, vegetable stock (onion powder, carrot extract, tomato powder, dried lovage, dried thyme, dried bay leaf), flavouring), dried egg yolk, spices (contains mustard), stabiliser (xanthan gum).
Supermarket Brand Lasagne	British Beef (20%), Whole Cows' Milk**,** Cooked Free Range Egg Pasta (16%) (Durum Wheat Semolina, Water, Whole Egg**,** Egg White), Tomato, Water, Tomato Purée, Onion, Red Wine (4%), Mushroom, Carrot, Mature Cheddar Cheese (2%) (Cows' Milk**)**, Cornflour, Garlic Purée, Fortified Wheat Flour (Wheat Flour, Calcium Carbonate, Iron, Niacin, Thiamin), Rapeseed Oil, Salt, Rosemary, Black Pepper, Oregano, Nutmeg, White Pepper, Bay Leaf.
